# Modeling and Optimal Input Design for Infra-Hepatic Blood Flow Regulation Systems

**DOI:** 10.3390/bioengineering13070749

**Published:** 2026-06-26

**Authors:** Yuxuan Huang, Zheng Zhang, Yi Duan, Hao Ye, Zhifeng Gao

**Affiliations:** 1 Department of Automation, Tsinghua University, Beijing 100084, China; hyx23@mails.tsinghua.edu.cn (Y.H.); haoye@tsinghua.edu.cn (H.Y.); 2 Department of Anesthesiology, Beijing Tsinghua Changgung Hospital, School of Clinical Medicine, Tsinghua Medicine, Tsinghua University, Beijing 102218, China; zzs01249@btch.edu.cn (Z.Z.); dya01610@btch.edu.cn (Y.D.)

**Keywords:** infra-hepatic inferior vena cava occlusion, balloon catheter, computational fluid dynamics, fluid–structure interaction, model predictive control, medical device control

## Abstract

Infra-hepatic inferior vena cava (IVC) balloon occlusion is an effective strategy for reducing intraoperative bleeding during precision liver surgery, yet rapid balloon inflation can produce abrupt transient deviations in downstream venous pressure that are not yet quantitatively characterized. Current practice relies on operator experience, with no quantitative framework to balance occlusion efficacy against downstream pressure safety. A computational fluid dynamics (CFD) model of the balloon-occluded IVC was developed in ANSYS 2025 R2 with two-way fluid–structure interaction (FSI), Carreau–Yasuda blood rheology, and a balloon described by an Ogden hyperelastic model; the flow regime was laminar (Re ≈ 254). Reduced-order ARX models of four input–output subsystems were identified from CFD-generated data, and a model predictive control (MPC) strategy was formulated to penalize downstream pressure overshoot through a weighted cost function. The identified models achieved training normalized root-mean-square errors of 0.0363 to 0.1164 and out-of-sample validation errors of 0.1224 to 0.2381. Conventional sigmoid inflation induced a 45.82% overshoot in downstream pressure (*P*_aft_); the optimal input signal (q = [0, 1, 0, 0], λ = 0.1) reduced this to 6.05%, a reduction of 39.77 percentage points, while preserving >90% flow occlusion at *U*_F_ = 3 × 10^4^ Pa. The proposed framework offers a quantitative basis for balloon-occlusion device design that limits downstream pressure overshoot, motivating subsequent benchtop, ex vivo, and in vivo validation.

## 1. Introduction

Precision liver resection depends on a near-bloodless surgical field, and infra-hepatic inferior vena cava (IVC) balloon occlusion is an attractive way to limit hepatic outflow bleeding because the degree of occlusion can be adjusted continuously through the catheter. The clinical difficulty is that rapid balloon inflation drives abrupt transient swings in downstream venous pressure, and the inflation rate is currently chosen from operator experience, with no quantitative framework that weighs occlusion efficacy against downstream pressure overshoot. The present work addresses this gap in silico, as a first-stage methodological proof of concept. Bleeding control in precision liver resection [1,2] relies mainly on inflow occlusion by the Pringle maneuver [3], yet this approach risks hepatic ischemia–reperfusion injury [4] and leaves hepatic-vein backflow uncontrolled, a major source of intraoperative blood loss [5]. Adding outflow occlusion lowers central venous pressure and reduces blood loss [3,6,7], an approach termed total hepatic vascular exclusion [5], and clamping the infra-hepatic IVC below the liver is an effective way to limit hepatic outflow [8,9]. Because current infra-hepatic IVC occlusion relies on external clamping [10,11], which can injure the vessel wall and is not always feasible [12,13], a safer and continuously adjustable occlusion technique is needed.

Alternatively, the balloon flotation catheter is a commonly used technique in clinical practice, with the advantage of safety and easy operation [14,15]. Internal occlusion by balloon catheter provides an alternative approach to caval occlusion [16] and allows continuous adjustment of the occlusion degree compared with external clamping. However, there is a lack of research on infra-hepatic IVC occlusion based on balloon catheter, except for a study by Zhu et al. [17], where a specially designed balloon catheter can effectively occlude the hepatic outflow in experiments on pigs with fixed inflation degrees.

CFD-based hemodynamic modeling has been widely applied to characterize venous flow patterns and inform vascular device design. Li et al. [18] modeled the hemodynamics of IVC filters using a Carreau–Yasuda blood rheology [19]; although not specific to the balloon-occluded IVC, such work informs the geometry and parameter choices adopted here.

As a mechanism model, the CFD simulation captures the underlying fluid dynamics but is too complex and computationally slow to serve directly as the control model needed for control-algorithm and input-signal design. System identification addresses this by fitting a simplified model to the input–output data generated by CFD simulation, yielding a model suitable for control [20]. The same strategy of pairing CFD simulation with identification-based predictive control has been applied in prior work [21].

MPC predicts the future output of a system under a candidate control sequence and selects the sequence that minimizes the deviation from the desired output [22]. Despite wide use in process and motion control, its applications in the medical field remain limited; one example is an MPC-based pressure controller for lung ventilation that accounts for patient-safety limits [23]. Combining CFD and MPC [21], and using MPC to weigh occlusion performance against blood pressure safety [23], motivates the present approach, in which the control model is obtained by system identification from CFD simulation data.

Three specific gaps remain at the intersection of these research strands. First, the available evidence on balloon-occluded infra-hepatic IVC is limited to static or fixed inflation states. Zhu et al. [17] demonstrated effective hepatic outflow occlusion in porcine experiments at preset inflation degrees, but the transient hemodynamic response during the dynamic inflation phase, which determines whether downstream pressure remains within safe physiological bounds, has not been quantitatively characterized. Second, THE existing CFD literature on inferior vena cava hemodynamics has focused predominantly on filter-related applications [18,24,25,26], whereas the coupled fluid–structure interaction generated by an inflating balloon catheter occluding the IVC has not been systematically modeled, leaving the relationship between input pressure trajectory and downstream pressure response largely unquantified. Third, clinical balloon inflation rates in current practice are guided by operator experience and qualitative protocols rather than by quantitative control logic; no formal input-design framework exists to balance the competing objectives of effective flow occlusion and downstream pressure safety. Addressing these gaps requires an integrated approach that couples high-fidelity CFD-FSI modeling of the dynamic balloon-occlusion process with control-oriented system identification and predictive input design.

To quantitatively analyze the dynamics of the occlusion process and design the optimal input pressure signal applied to the balloon, this paper focuses on the modeling, simulation, and control of the infra-hepatic IVC occlusion process using a balloon catheter.

A high-fidelity computational fluid dynamics (CFD) model of the balloon-occluded inferior vena cava (IVC) system is developed using ANSYS. The model captures the coupled dynamics between balloon deformation and blood flow, enabling systematic characterization of hemodynamic responses during controlled vascular occlusion.

CFD simulations reveal a fundamental trade-off in balloon–catheter control: while internal balloon pressure achieves effective vascular occlusion, it concurrently induces transient downstream pressure perturbations. These fluctuations characterize the dynamic overshoot during inflation and motivate an input-design objective that limits it. Accordingly, downstream pressure overshoot *P*_aft_ is treated as the primary penalized objective in the control cost function.

Building on this analysis, a model predictive control (MPC) framework is proposed to shape balloon inflation by penalizing downstream pressure overshoot through a weighted cost function. The control signal design method trades off occlusion efficacy against downstream pressure overshoot, enabling systematic, reproducible modulation of the balloon–catheter system.

The remainder of the paper presents the numerical methodology (Section 2), the simulation results (Section 3), the discussion and clinical implementation considerations (Section 4), and the conclusions (Section 5).

## 2. Numerical Simulation Methodology

### 2.1. Theoretical Preliminaries: Generalized Predictive Control

In this work, the Generalized Predictive Control (GPC) algorithm, one of the classic MPC algorithms based on the stochastic discrete-time model, is used to design the optimal input signal. The necessary notations and calculation steps are as follows.

Suppose the system to be controlled can be described by an ARX model [27]:

(1) $$A\left(z^{-1}\right)y(k)=B\left(z^{-1}\right)u(k-1)+e(k)$$

where *u*(*k*) and *y*(*k*) are the system input and output at time step *k*, respectively, *e*(*k*) is the unknown white noise, *z*^−1^ denotes the delay operator, and

(2) $$A\left(z^{-1}\right)=1+a_{1}z^{-1}+a_{2}z^{-2}+\cdots +a_{n}z^{-n},B\left(z^{-1}\right)=b_{0}+b_{1}z^{-1}+b_{2}z^{-2}+\cdots +b_{n}z^{-n}$$

Before introducing the GPC law, the following notations need to be defined [28]: Let *G*(*z*) = *B*(*z*^−1^) *A*^−1^(*z*^−1^) = *g*_1_*z*^−1^ + *g*_2_*z*^−2^ + … denote the transfer function from *u* to *y*, and let *E_j_* and *F_j_* represent the solutions of the so-called Diophantine equation 1 = *E_j_*(*z*^−1^)*A*Δ + *z*^−1^ *F_j_*(*z*^−1^), where Δ = 1 − *z*^−1^ represents the difference operator, *j* is the prediction length. Define *G_j_* = *E_j_ B* = *g_j_*_,0_ + *g_j_*_,1_ *z*^−1^ + … + *g_j_*_,*n*+*j*−1_*z*^−(*n*+*j*−1)^, where *g_j_*_,*i*_ = *g_i_*_+1_(*i* < *j*), and define matrix *G* and vector *f* appropriately (see [28] for details).

Let *ŷ*(*k* + *j*) denote the prediction of the system output at time step *k* + *j*, which can be calculated by [28]:

(3) $$\hat{y}(k+j)=G_{j}\left(z^{-1}\right)\Delta u(k+j-1)+F_{j}\left(z^{-1}\right)y(k)$$

Then the GPC determines the optimal input sequence at each time step *k* by solving the following optimization problem [28]:

(4) $$J=\sum_{j=N_{1}}^{N_{2}}{\left[\hat{y}(k+j)-\omega (k+j)\right]}^{2}+\lambda \sum_{j=1}^{NU}{\left[\Delta u(k+j)\right]}^{2}$$

where the first summation term in *J* describes the errors between the predesigned reference trajectory ω and the predicted output signal y in the time duration from *k* + *N*_1_ to *k* + *N*_2_, and the second one penalizes the amplitude variations in the input signal in the time duration from *k* + 1 to *k* + *NU*. Parameter λ is a weight coefficient to trade off the two kinds of objectives.

According to [28], at time step *k*, the optimal control law obtained by solving Equation (4) is:

(5) $$U={\left(G^{T}G+\lambda I\right)}^{-1}G^{T}\left(\omega -f\right)$$

where *U* = [Δ*u*(*k* + 1), Δ*u*(*k* + 2), …, Δ*u*(*k* + *NU*)]*^T^*, *ω* = [*ω*(*k* + *N*_1_), …, *ω*(*k* + *N*_2_)]*^T^*.

### 2.2. Problem Formulation

**Figure 1:**
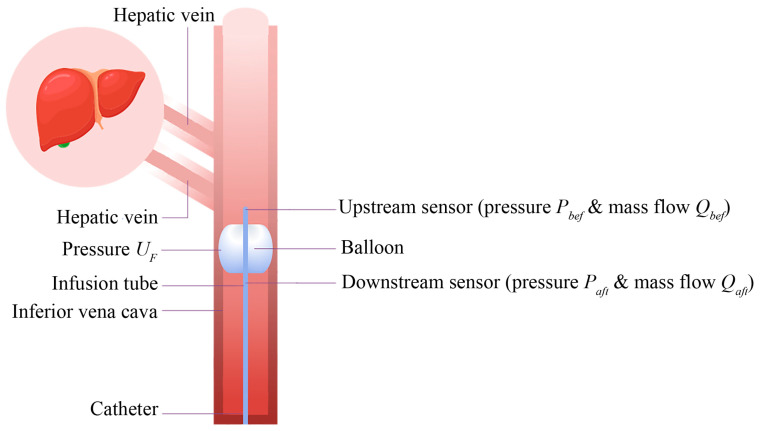
Schematic of liver outflow occlusion using a balloon catheter.

The schematic of the liver outflow occlusion system considered in this paper is shown in Figure 1, where the occlusion of the infra-hepatic IVC blood is controlled by the inflation of the balloon, which is further controlled by the pressure *U*_F_ exerted on the balloon’s inner wall. It is assumed that *U*_F_ can be manipulated, and the blood pressures and flow rates of the IVC upstream and downstream of the balloon can be measured online. Let *P*_bef_ and *Q*_bef_ denote the upstream pressure and flow rate, respectively, while *P*_aft_ and *Q*_aft_ denote the downstream pressure and flow rate, respectively. Intuitively, the dynamics of the occlusion process can be regarded as a dynamical system with *U*_F_ as input, which can be manipulated, and with *P*_bef_, *P*_aft_, *Q*_bef_, and *Q*_aft_ as outputs, which include the flow rates to reflect the occlusion extent and the blood pressures to reflect clinically relevant fluctuations, since abrupt changes in *P*_aft_ may signal localized hemodynamic disturbances and are considered undesirable during liver resection.

In clinical use, the operator advances the balloon catheter through the vasculature into the infra-hepatic IVC and inflates the balloon to occlude hepatic venous return during resection. Because the degree of occlusion is adjusted continuously through the catheter, the operator can tighten or ease the occlusion as the surgical field requires. The present framework supports this workflow by computing the optimal inflation-pressure input *U*_F_ that drives the balloon toward the target occlusion while limiting the downstream pressure overshoot, replacing experience-based inflation with a reproducible input trajectory.

In the following subsections, we first build a simulation model of the IVC occlusion system shown in Figure 1, based on the CFD software ANSYS, and present simulation experiments to show that although the blood flow can be successfully occluded, there are pronounced transient pressure fluctuations. Then, we identify four simplified control models suitable for MPC by fitting the simulation data generated by the CFD model, and further design an optimal input pressure signal *U*_F_ to balance effective occlusion against the magnitude of the downstream pressure transient.

### 2.3. CFD-FSI Simulation Model of the Balloon-Occluded IVC System

Since the inflation and deflation of the balloon will lead to large deformation of the geometric structure of the solid model on one hand, and impact the fluid dynamics within the IVC blood flow on the other hand, the two-way fluid–structure interaction (FSI) analysis [29,30] is employed in the simulation modeling of the system, which simultaneously considers the influence on the solid caused by the deformation of the fluid and the influence on the fluid caused by the deformation of the solid, particularly effective in solving the problem associated with large deformation.

Two-way FSI was adopted because the degree of occlusion is an output of the simulation rather than a quantity that can be prescribed. The control input in this study is the pressure *U*_F_ applied to the balloon inner wall, and the balloon expansion, and hence the occlusion of the IVC, must be solved as the coupled mechanical and hydrodynamic response to that pressure. The motion of the balloon wall is therefore not known in advance and cannot be specified beforehand. Two-way FSI resolves the large-deformation response of the balloon, represented by a third-order Ogden hyperelastic model, together with the interface tractions exchanged across the fluid-solid boundary, so that the balloon deformation and the downstream pressure both emerge as responses to the applied pressure. A cylindrical-expansion approach using dynamic mesh layering and remeshing typically requires the wall kinematics to be prescribed in advance. Even when the kinematics are driven by a force balance through a user-defined function or a six-degree-of-freedom solver, this approach would impose the occlusion trajectory rather than predict it, and it would not resolve the balloon’s hyperelastic stress–strain response or recover the pressure-to-occlusion mapping on which the input-design framework depends.

#### 2.3.1. Geometric Model

**Figure 2:**
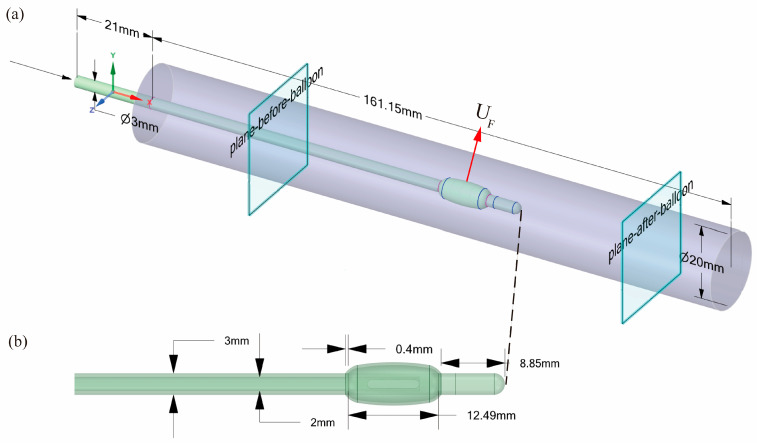
The geometric model of the balloon-occluded IVC system in ANSYS. (**a**) The geometric model and dimension sizes of IVC. Two data collection planes and the system input *U*_F_ are labeled in the figure. (**b**) The geometric model and dimension sizes of the balloon catheter.

Figure 2a shows the geometry of the IVC model considered in this study. The blood flow within the IVC is modeled as a cylindrical fluid domain with a diameter of 20 mm, which lies within the physiological range of adult infrarenal IVC diameters reported in ultrasound and MRI studies (typically 17–25 mm) in patients under general anesthesia or controlled ventilation [31]. Because the IVC diameter varies dynamically with respiration, the present model represents an averaged quasi-end-expiratory state rather than the full spectrum of respiration-induced variability [18,24]. The length of the fluid domain is set to approximately 161 mm to ensure sufficient upstream and downstream space for the flow to reach a quasi-steady state around the balloon.

The IVC wall is modeled as a rigid boundary in the present study. This assumption is adopted to simplify the computational model and to improve numerical stability and convergence in the two-way FSI analysis. The primary objective of this work is to evaluate the interaction between blood flow and the deformable balloon; introducing an elastic vessel wall as an additional deformable component would substantially increase computational cost and convergence difficulties. A rigid-wall representation also neglects venous compliance, which in vivo tends to buffer part of the pressure rise. Consequently, the present CFD model is expected to overestimate the amplitude of transient downstream pressure changes compared with physiologically compliant veins. In this sense, the rigid-wall simulations can be interpreted as a conservative scenario with respect to pressure overshoot.

The solid domain of the system consists of a balloon and a catheter, whose local geometric model and dimension sizes are illustrated in Figure 2b. The balloon is modeled as a hollow ellipsoidal structure, with a catheter passing horizontally through its center. The thickness of the balloon wall is set to 0.4 mm. To achieve complete occlusion of the IVC during the inflation of the balloon, the major axis of the balloon’s inner wall is set to 12.49 mm. The catheter has outer and inner diameters of 3 mm and 2 mm, respectively. Its head extends 8.85 mm behind the balloon. The catheter is equipped with two openings, allowing infusion of fluid into the interior of the balloon.

#### 2.3.2. Mesh Generation and Quality Assessment

The two-way fluid–structure interaction analysis was performed using ANSYS 2025 R2. The fluid and structural domains were discretized according to their respective governing equations: the finite volume method (FVM) was used for the fluid domain (ANSYS Fluent), while the finite element method (FEM) was used for the structural domain (ANSYS Mechanical) [32,33]. The two solvers were coupled at every coupling step through ANSYS System Coupling, which handled the bidirectional exchange of interface displacements (FEM to FVM) and traction loads (FVM to FEM) at the balloon outer wall. Internal subiterations were performed within each coupling step until interface force and displacement residuals satisfied preset convergence tolerances.

A hybrid meshing strategy was adopted to balance fidelity at the deforming balloon-fluid interface against computational cost in the bulk venous lumen. The fluid region immediately surrounding the balloon was discretized with unstructured tetrahedral elements to accommodate the large local geometric changes imposed by balloon inflation, whereas the upstream and downstream straight portions of the IVC were discretized with structured hexahedral elements, which better preserve the cylindrical geometry and reduce numerical diffusion in regions of approximately parallel flow. Local mesh refinement was applied at the fluid-solid interface to ensure high-fidelity force and displacement mapping in the narrow clearance region around the inflated balloon. For the medium mesh adopted in the balloon occlusion experiment in Section 2.4, the maximum cell skewness was 0.857 and the minimum orthogonal quality was 0.143. Grid-independence is further verified in Section 2.3.5.

#### 2.3.3. Material Properties and Boundary Conditions

The fluid and solid properties of the ANSYS model for simulations are set as follows. A non-Newtonian formulation was adopted because the Newtonian assumption can appreciably bias hemodynamic predictions relative to shear-thinning models in comparable vascular CFD settings [34]. The shear-thinning properties of human blood can be described by the Carreau–Yasuda model [18,24], which describes the non-linear relationship between blood viscosity *μ* and shear rate *γ* as follows [19]:

(6) $$\mu (\gamma )=\mu_{\infty }+\left(\mu_{0}-\mu_{\infty }\right){\left[1+{\left(\lambda \gamma \right)}^{2}\right]}^{\frac{n-1}{2}}$$

where *n* = 0.3568, *λ* = 3.313 s, *μ*_∞_ = 0.00345 Pa·s, *μ*_0_ = 0.056 Pa·s. According to [18], the blood density is set as *ρ*_b_ = 1060 kg/m^3^. The third-order Ogden hyperelastic model was used to represent the large deformation of the balloon during inflation, accommodating strains of up to approximately 700% [35]. The Ogden material parameters were obtained by fitting the experimental stress–strain data available in the ANSYS Mechanical material library. The deviatoric behavior is defined by *μ*_1_ = 41.76 Pa, *α*_1_ = 7.396; *μ*_2_ = 41.766 Pa, *α*_2_ = 7.4431; and *μ*_3_ = 41.766 Pa, *α*_3_ = 7.443. To enforce near-incompressibility, the volumetric parameters were set to *D*_1_ = *D*_2_ = *D*_3_ = 0 Pa^−1^, corresponding to an effective Poisson’s ratio approaching 0.5.

To prescribe the inflow-outflow condition, a relative pressure difference of 30 Pa (approximately 0.22 mmHg, using 1 mmHg = 133.32 Pa) is imposed between the inlet and outlet of the IVC blood domain. This pressure scale is illustrative: it was chosen for numerical stability and to demonstrate the input-design method, and the resulting absolute values, including the steady-state downstream pressure of approximately 0.399 Pa (approximately 0.003 mmHg) reported in Section 2.3.5, lie well below physiological venous pressures and are not intended to represent in vivo absolute levels. The quantity of interest in this study is therefore the relative suppression of downstream pressure overshoot rather than the absolute pressure magnitude. Because the model is nonlinear and these simulations run at the current low-pressure operating point, the reported overshoot behavior, including both the absolute pressures and the relative suppression, is specific to this operating point and to the present grid resolution. This study does not claim that these magnitudes transfer unchanged to physiological pressure and flow, where the Reynolds number, the jet and pressure-recovery structure, and the fluid–structure coupling may all change; confirming this behavior at physiological scale is left to future benchtop and in vivo studies. The contribution of this study is the input-design methodology itself, demonstrated in silico at this operating point. Under this configuration, the resulting simulated mass flow rate is approximately 0.04 kg/s (corresponding to ≈2.0 L/min) [36], which lies within the physiological range of venous return reported for adult patients at rest or under general anesthesia. During spontaneous breathing, IVC flow exhibits pronounced respiratory modulation; however, the present model adopts a steady inflow condition to represent an averaged or breath-hold intraoperative state, which is commonly used in first-stage CFD and methodological evaluations.

The complete set of boundary conditions for the coupled simulation is summarized as follows. Balloon inflation was produced by a pressure load applied to the balloon inner wall, and this applied pressure served as the system input *U*_F_; the balloon expansion and the resulting IVC occlusion emerged from the two-way coupling rather than being prescribed beforehand. Applying the load directly to the inner wall reproduced the expansion obtained by infusing fluid through the catheter while reducing the computational cost. The IVC wall was treated as a rigid boundary, as described in Section 2.3.1, and a no-slip condition was imposed on all wetted solid surfaces, including the IVC wall and the balloon outer wall. The inlet-outlet pressure difference of 30 Pa specified above completed the flow boundary conditions, and the fluid-solid interface at the balloon outer wall was resolved through the two-way coupling described in Section 2.3.2. The CFD solver advanced with a fixed computational time step of 0.01 s in Section 2.4, which set the interval at which the governing equations were integrated and the simulated input and output signals were recorded. In Section 2.5, to balance computational efficiency and model fidelity, the CFD computational time step was set to 0.1 s during the identification data collection, which lasts 250 s. A larger interval, Δ*t* = 0.5 s, was used to sample the input and output data for system identification (Section 2.5). The CFD computational time step for both the heuristic, non-optimal and optimal input signal experiments (Section 2.7) was set to 0.1 s to maintain consistency with the identification model.

Gravitational effects were not included in the present simulations. Although the imposed inlet-outlet pressure difference of 30 Pa corresponds to a hydrostatic head of approximately 3 mm in blood, the simulated IVC segment (161 mm in length) was modeled in the horizontal orientation, consistent with the supine intraoperative posture during hepatic resection. Under this configuration, the contribution of gravity to axial flow is negligible compared with the balloon-induced transient pressure dynamics that are the primary focus of this study.

#### 2.3.4. Reynolds Number Verification

The flow regime within the simulated IVC was verified by computing the Reynolds number under the baseline boundary conditions. The global Reynolds number is defined as

(7) $$Re=\frac{\rho \bar{u}D}{\mu_{a}}$$

where *ρ* = *ρ*_b_ = 1060 kg/m^3^ is the blood density, *ū* ≈ 0.12 m/s is the mean axial velocity derived from the simulated mass flow rate of approximately 0.04 kg/s through a circular cross-section of diameter *D* = 0.02 m, and *μ*_a_ denotes the apparent dynamic viscosity. Because blood is modeled as a shear-thinning Carreau–Yasuda fluid, *μ*_a_ varies spatially with the local shear-rate field; in this study *μ*_a_ ≈ 0.01 Pa·s was taken as the spatially averaged dynamic viscosity computed from the converged Carreau–Yasuda viscosity field across the IVC cross-section. The resulting global Reynolds number, Re ≈ 254, lies well below the laminar-turbulent transition threshold for circular pipe flow (Re ≈ 2300), confirming that a laminar flow assumption is appropriate for the present simulations.

At the maximum balloon expansion state, the severe constriction imposes substantial flow resistance, leading to physiological flow suppression. Consequently, the local mass flow rate drops significantly, and the global maximum velocity in the fluid domain (about 0.064 m/s) falls well below the baseline mean axial velocity (0.12 m/s). With both the local velocity and the local hydraulic length scale substantially reduced, the local Reynolds number at maximum occlusion decreases even further below the global value. The global laminar assumption therefore remains robustly valid under the worst-case constriction encountered in the present simulations.

#### 2.3.5. Mesh Independence Verification

A grid refinement study was conducted to quantify the spatial discretization error following the grid convergence index (GCI) methodology [37]. For this study, the same input signal (*U*_F_) as in the balloon occlusion process (Section 2.4) was applied. Three mesh densities were evaluated: a coarse mesh (1.16 × 10^5^ elements), a medium mesh (3.34 × 10^5^ elements), and a fine mesh (1.03 × 10^6^ elements). Because the principal output of interest in this study is the downstream pressure response, the steady-state downstream pressure *P*_aft_ at full balloon expansion was selected as the representative scalar for convergence analysis. The corresponding values of *P*_aft_ and the GCI between successive mesh refinements are summarized in Table 1.

Mesh refinement produced a monotonic decrease in *P*_aft_ from 0.494 Pa (coarse) to 0.412 Pa (medium) to 0.399 Pa (fine). The GCI between the medium and fine meshes was 0.66%, indicating that the medium mesh is acceptably converged with respect to *P*_aft_ for the present study. To balance computational cost against discretization accuracy, the medium mesh was adopted in subsequent balloon occlusion simulations.

A temporal time-step independence study complemented the spatial grid-convergence analysis. The inflation simulation was repeated at solver time steps of 0.02, 0.01, and 0.005 s, and the peak transient downstream pressure, the inflation-induced trough in *P*_aft_, reached −6.424, −6.443, and −6.459 Pa, respectively. Refining the step from 0.02 to 0.005 s changed the peak by less than 0.9%, confirming that the 0.01 s production step resolves the peak transient (Figure S1).

The peak transient of *P*_aft_ across the coarse, medium, and fine meshes reached −3.012, −6.443, and −7.629 Pa, respectively, without reaching an asymptotic convergence on the meshes, consistent with an under-resolved local feature in the high-gradient region near the balloon during rapid inflation. We do not claim a grid-converged absolute value for the peak transient. However, this will not influence the conclusion that our proposed method can effectively suppress the overshoot of *P*_aft_, because the identification process, the comparative experiments of baseline and optimized inputs (Section 2.5 and Section 2.7) are all computed on the simplified CFD model with same mesh (See Section 2.4), their comparison is like-for-like, and the reported quantity is the relative suppression of the overshoot rather than the absolute peak. Grid-converged transient prediction, using finer or adaptively refined meshes with Richardson extrapolation, is identified as future work (Section 6).

### 2.4. CFD Simulation of the Balloon Occlusion Process

To capture the changes in the system variables during the blood occlusion process, we conducted an experiment to simulate the balloon occlusion process in the IVC based on the CFD simulation model. As shown in Figure 2a, two data collection planes with coordinates at *x* = 46 mm and *x* = 150 mm are set at upstream and downstream of the balloon, respectively, to save the output signals *P*_bef_, *P*_aft_, *Q*_bef_, and *Q*_aft_ on the cross-section of the blood flow.

The calculation time step in the experiment, which is also the CFD sampling time for input and output signals, was set to 0.01 s. The simulation experiments last for 30 s.

**Figure 3:**
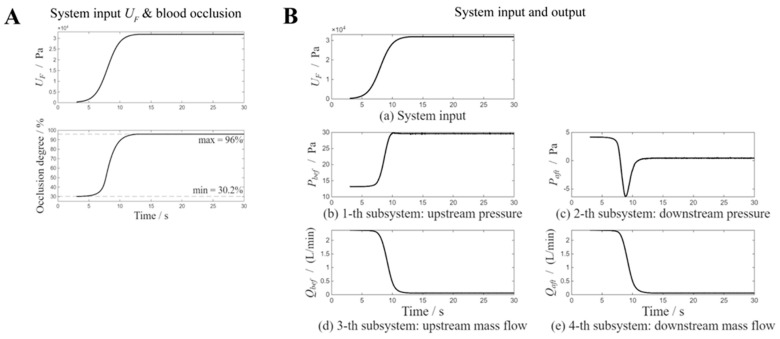
Results of blood occlusion simulation experiments. (**A**) System input *U*_F_ and the blood occlusion degree. (**B**) System input and output in the experiment. Subfigures (**a**,**b**) display the system input signal, while subfigures (**c**–**e**) show the output signals of the four subsystems.

Figure 3A reveals that the occlusion degree in the IVC gradually increases from 30% to about 96% with the increase in the system input *U*_F_ from 0 to 3.2 × 10^4^ Pa. The input pressure *U*_F_ is simulated as a sigmoid function.

**Figure 4:**
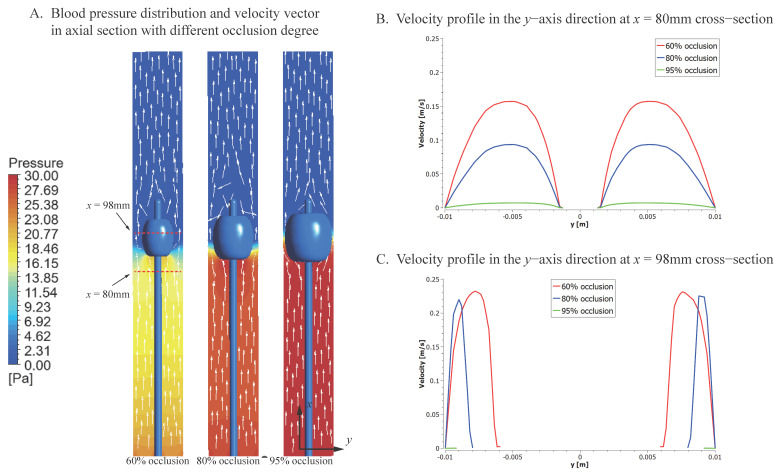
Hemodynamic response of the infra-hepatic IVC at three balloon occlusion degrees (60%, 80%, 95%). (**A**) Axial-section blood pressure distribution overlaid with velocity vectors at the three occlusion states. (**B**) Velocity profile along the *y*-axis at *x* = 80 mm (upstream cross-section). (**C**) Velocity profile along the *y*-axis at *x* = 98 mm (narrowest cross-section of the balloon occlusion).

Figure 4A presents the axial-section pressure distribution overlaid with velocity vectors within the infra-hepatic IVC at three representative balloon occlusion degrees (60%, 80%, and 95%). As the occlusion degree increases, the upstream-to-downstream pressure transition across the balloon steepens progressively and the velocity field concentrates within the narrowing residual gap between the balloon outer wall and the IVC wall. The velocity profiles along the y-axis at two axial positions are compared in Figure 4B,C, where *x* = 80 mm is located upstream of the balloon and *x* = 98 mm corresponds to the narrowest cross-section of the balloon occlusion. In the upstream region (Figure 4B), each profile exhibits a classic annular flow distribution due to the presence of the catheter, with the velocity going to zero at both the vessel wall and the catheter surface. At the maximum occlusion site (Figure 4C), the flow within the residual gap is progressively suppressed as the occlusion degree increases from 60% to 95%: the increasing constriction raises the local flow resistance and lowers the gap velocity rather than forming a high-speed jet, with the global maximum velocity in the fluid domain falling to about 0.064 m/s at 95% occlusion, below the baseline mean axial velocity of 0.12 m/s. The progression from Figure 4A–C illustrates how increasing balloon expansion shifts the system from a uniformly perfused state toward a constriction-dominated regime in which the downstream pressure response is governed by the post-balloon pressure-recovery dynamics, motivating the input-design framework developed in subsequent sections.

The system input and its output signal response (i.e., the pressures *P*_bef_ and *P*_aft_, and the flow rates *Q*_bef_ and *Q*_aft_ on the data collection planes) are plotted together in Figure 3B, where the raw mass flow rate mf (in kg/s) collected in the CFD model is converted to volumetric flow rate *Q* (in L/min). As shown in Figure 3B, the flow rate decreases sharply in the occlusion process. Also, the pressure upstream of the balloon, the flow rates upstream and downstream of the balloon (i.e., *P*_bef_, *Q*_bef_, and *Q*_aft_) track the input signal without pronounced fluctuations. However, the pressure downstream of the balloon (i.e., *P*_aft_) shows a noticeable overshoot as the input pressure increases. The simulation results in this section indicate that:

(i) The balloon inflation controlled by the input signal *U*_F_ can efficiently block the IVC blood;
(ii) During the occlusion process, an overshoot is observed in the pressure downstream of the balloon (i.e., *P*_aft_), indicating an abrupt transient deviation in downstream venous pressure. Such rapid fluctuations may contribute to localized hemodynamic perturbations, which are clinically undesirable during major liver surgery.

Therefore, the main objective of the input signal design in the next subsection is twofold: to block the flow in the IVC and to suppress the overshoot of *P*_aft_.

**Remark** **1.** *Given that the primary object of this study is to validate the feasibility and effectiveness of the proposed control framework, a simplified CFD model was employed for the subsequent identification and control experiments to optimize computational efficiency. While a systematic offset in absolute magnitude is observed relative to the high-fidelity baseline, the dynamic evolution of the flow field remains highly consistent, providing a robust basis for control validation. The mesh quality metrics for this simplified model, which comprises approximately 6.3 × 10^4^ elements, are summarized as follows: the maximum cell skewness was 0.764, the minimum orthogonal quality was 0.236, and the maximum aspect ratio was 8.747, all of which lie within commonly accepted ranges for hybrid FVM-FEM FSI simulations of similar geometric complexity [32]. The slightly elevated skewness values are concentrated in the narrow annular gap between the balloon outer wall and the IVC wall, where geometric constraints unavoidably distort local element shape; these high-skewness elements remain confined to the balloon-fluid interface region and do not propagate into the bulk flow domain.*

### 2.5. Control Model Identification for MPC

As previously mentioned, due to its high complexity and slow computation speed, the CFD model cannot be used as a control model for the MPC-based input signal design. So, in this subsection, the low-order control models for the four balloon-occluded IVC subsystems between the input signal *U*_F_ and the output signals *P*_bef_, *P*_aft_, *Q*_bef_, and *Q*_aft_ were built by least-squares (LS) method for dynamical system identification [27].

Since the system has only one input, i.e., *U*_F_, but four outputs, it can be regarded as four single-input single-output (SISO) subsystems. For convenience, throughout the paper, the subsystems with *P*_bef_, *P*_aft_, *Q*_bef_, and *Q*_aft_ as outputs are called subsystems 1, 2, 3, and 4, respectively.

Suppose the four subsystems can be described by four ARX models as introduced in Section 2.1. Following the predefined notation, let *u*(*k*) denote the input pressure *U*_F_ added to the balloon’s inner wall in the CFD model at time instant *k*, and *y_i_*(*k*) (for *i* = 1, 2, 3, 4) denote the measurement of *P*_bef_, *P*_aft_, *Q*_bef_, and *Q*_aft_ at time instant *k*, respectively. For subsystem *i* (for *i* = 1, …, 4), let *ŷ_i_*(*k*) denote the estimated output, *h_i_*(*k*) = [−*y_i_*(*k* − 1) ⋯ −*y_i_*(*k* − *n*) *u*(*k* − 1) ⋯ *u*(*k* − *n*)]^T^ denote the vector composed of the lagged inputs and outputs, *A_i_*(*z*^−1^) and *B_i_*(*z*^−1^) denote the ARX polynomials, and *θ_i_* represent the parameter vector composed of the coefficients in *A_i_*(*z*^−1^) and *B_i_*(*z*^−1^). Then the LS estimation of *θ_i_* is given by [27]:

(8) $$\hat{\theta_{i}}={\left(\sum_{k=1}^{L}h_{i}(k)h_{i}{\left(k\right)}^{T}\right)}^{-1}\sum_{k=1}^{L}h_{i}(k)y_{i}(k)$$

which is the optimal solution of

(9) $$J\left(\theta_{i}\right)=\sum_{k=1}^{L}{\left[y_{i}(k)-h_{i}^{T}(k)\theta_{i}\right]}^{2}$$

with

(10) $$h_{i}(k)={\left[-y_{i}(k-1),\cdots ,-y_{i}(k-n),u(k-1),\cdots ,u(k-n)\right]}^{T},\theta_{i}={\left[a_{i,1},\cdots ,a_{i,n},b_{i,1},\cdots ,b_{i,n}\right]}^{T}$$

From Equation (8), it can be seen that the calculation of the model parameters *θ_i_* requires the input and output data *u*(*k*) and *y_i_*(*k*), for *k* = 1, …, *L*, *i* = 1, …, 4. In this paper, we generated the output data using the CFD simulation model established in the previous subsection with a given input signal.

**Remark** **2.** *Building models as Equation (9) through fitting the data generated by the CFD model has the following benefits: (i) The models in the form of Equation (9) are much simpler than the CFD model, and therefore can be used as a model required by MPC when designing the optimal input signal; (ii) Since the models are estimated by fitting the data generated by the CFD model, for the same input, they can give similar estimated outputs as the CFD model, or in other words, the simplified control models and the CFD model are equivalent in terms of input–output relation.*

**Remark** **3.** *To guarantee the persistent excitation of the input signal, which is important to ensure identifiability [27], the so-called M-sequence input [27] was designed as the system input U_F_ in this paper. The input and output data U_F_, P_bef_, P_aft_, Q_bef_, and Q_aft_ obtained from the simulation experiment were first zero-centered and then sampled at intervals of Δt = 0.5 s.*

Through elbow analysis [27], the order of each control model of the subsystems was chosen to be two (i.e., there are two parameters to be estimated in both *A_i_*(*z*^−1^) and *B_i_*(*z*^−1^)), based on the declining trend of the calculated objective function with different n in Equation (8). The final results of the parameter identification are shown in Table 2. To evaluate the fitting accuracy of the training set, the normalized root mean square errors (NRMSE) of the four control models are calculated. The results show that the control models achieve satisfactory fitting accuracy and are suitable for the control of the IVC blood system. To assess the identified models on data not used for identification, an out-of-sample validation was performed by exciting the same two-way FSI CFD model with a sinusoidal input and comparing the one-step-ahead ARX prediction against the CFD response, with the signals zero-centered using the training-set parameters and sampled at Δ*t* = 0.5 s. The prediction reproduced the CFD response with R^2^ above 0.94 for all four subsystems (*P*_bef_ 0.9824, *P*_aft_ 0.9433, *Q*_bef_ 0.9850, *Q*_aft_ 0.9840), confirming that the linear structure captures the dominant dynamics over the operating envelope. The corresponding validation NRMSE values were 0.1326, 0.2381, 0.1224, and 0.1264 for *P*_bef_, *P*_aft_, *Q*_bef_, and *Q*_aft_, respectively, with *P*_aft_ remaining the most demanding subsystem, consistent with its strongly transient response. The validation curves are shown in Figure S2.

### 2.6. Optimal Input Signal Design Based on MPC

The aforementioned simulation experiment of the balloon occlusion process shows that the balloon inflation for IVC occlusion may cause an overshoot of *P*_aft_, the pressure downstream of the balloon, which represents a pronounced transient in the downstream pressure. To overcome the overshoot caused by blood occlusion, an optimal input signal design method for the system is proposed in this subsection based on the control model obtained before and the MPC method in Section 2.1.

#### 2.6.1. Objective Function Design for the Optimization Problem

According to Equation (3), the objective function of the input signal optimization for subsystem *i* (for *i* = 1, 2, 3, 4) considering both the errors between the predicted output *ŷ_i_* and the reference trajectories *ω_i_* during the time period from *k* + *N*_1_ to *k* + *N*_2_ and a penalty on the amplitude variation during the time period from *k* to *k* + *NU* of the input *U*_F_ can be written as:

(11) $$J=\sum_{i=1}^{4}q_{i}\sum_{j=N_{1}}^{N_{2}}{\left[\hat{y_{i}}(k+j)-\omega_{i}(k+j)\right]}^{2}+\lambda \;\sum_{j=1}^{NU}{\left[\Delta u(k+j)\right]}^{2}$$

where *ŷ_i_* for *i* = 1, 2, 3, 4 denote *P*_bef_, *P*_aft_, *Q*_bef_, and *Q*_aft_, respectively, and *ω_i_* denote the reference trajectories for the *i*-th subsystem outputs. The weight coefficients *q_i_* for *i* = 1, 2, 3, 4 are introduced to trade off the relative contribution of the tracking errors of the four subsystems. In addition, the parameters *N*_1_, *N*_2_, and *NU* were set as 3, 20, and 10, respectively, and the sampling time Δ*t* was set as 0.5 s.

#### 2.6.2. Reference Trajectory Design

**Figure 5:**
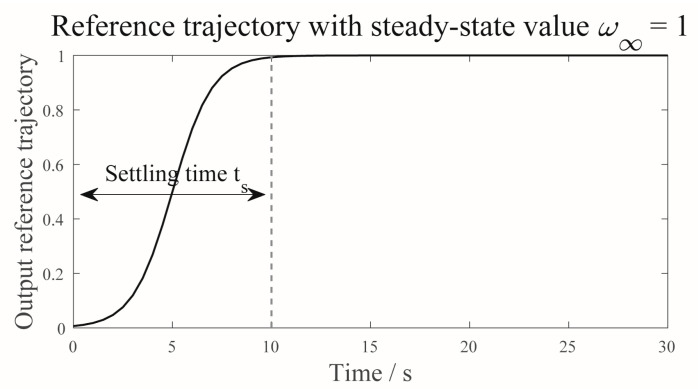
An example of the reference trajectory of the output signal with *ω*_∞_ = 1.

The reference trajectories for the output signal *P*_bef_, *P*_aft_, *Q*_bef_, and *Q*_aft_ in the four subsystems were designed such that: (i) overshoot and fluctuations in the transition process are minimized [38]; (ii) the steady-state values are consistent with the final values of the output signal as in the non-optimal input experimental results as shown in Figure 5; (iii) the settling time is set to match that of the input signal, balancing the system’s robustness and control speed [38].

**Figure 6:**
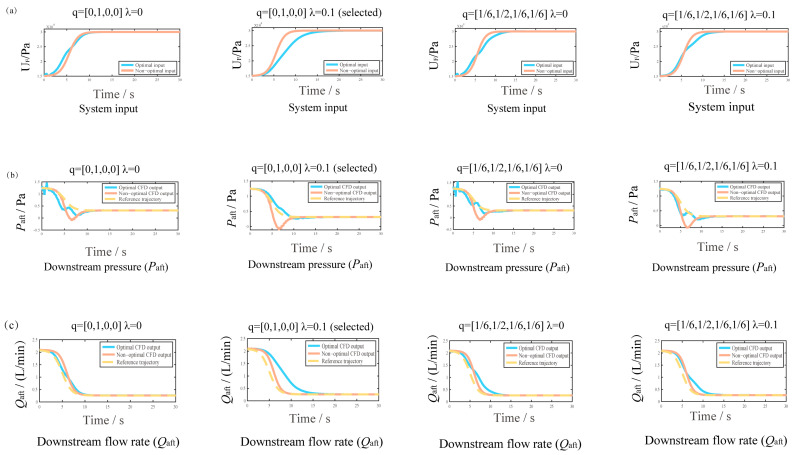
Effect of the cost-function weights q and λ on the optimal input design. Rows (**a**–**c**) present the system input UF, the downstream pressure Paft, and the downstream flow rate Qaft, respectively. From left to right, the four columns correspond to q = [0, 1, 0, 0], λ = 0; q = [0, 1, 0, 0], λ = 0.1; q = [1/6, 1/2, 1/6, 1/6], λ = 0; and q = [1/6, 1/2, 1/6, 1/6], λ = 0.1. The boxed second column (q = [0, 1, 0, 0], λ = 0.1) is the selected optimal setting. In each panel, blue and orange solid lines denote the optimal and non-optimal cases, respectively, corresponding to the input *U*_F_ in row (**a**) and to the CFD output in rows (**b**,**c**); the yellow dashed lines in rows (**b**,**c**) denote the reference trajectory. The non-optimal baseline (orange) is common to all four columns. The corresponding upstream pressure *P*_bef_ and upstream flow rate *Q*_bef_ are provided in Figures S3 and S4.

The *i*-th reference trajectory follows a sigmoidal (logistic) profile. Writing *τ* for the elapsed time from the start of the transition, it is given by

(12) $$\omega_{i}(\tau )=\frac{\omega_{i,\infty }}{1+exp\left(-5\left(\tau -t_{u}\right)/t_{u}\right)},\;i=1,2,3,4$$

where *ω_i_*_,∞_ represents the steady-state value of the reference trajectory and was set equal to the steady-state value of the output signal in the non-optimal input baseline, as shown in Figure 6. *t_u_* was set as 5 s, which is equal to the half of the settling time. To give an example, Figure 5 shows a typical reference trajectory in this work with a steady-state value *ω*_∞_ = 1.

#### 2.6.3. Solution of Optimal Input Signal

According to Equation (5), the following input increment matrix *U*_F_ at time step *k*:

(13) $$\Delta U_{F}(k)={\left(\sum_{i=1}^{4}q_{i}G_{i}^{T}G_{i}+\lambda I\right)}^{-1}\sum_{i=1}^{4}q_{i}G_{i}^{T}\left(\omega_{i}-f_{i}\right)$$

gives the optimal solution of Equation (10), where *G_i_*, *f_i_* represent the parameters *G*, *f* of the *i*-th subsystem and can be obtained from the ARX models as explained in Section 2.1. The final solution of *U*_F_(*k*) can be calculated by *U*_F_(*k*) = *U*_F_(*k* − 1) + Δ*U*_F_(*k*).

### 2.7. Validation Experimental Design

Simulation experiments were conducted to verify the proposed optimal input signal design method, in which the optimal input signal was solved based on the MPC algorithm, where the required control model was obtained through system identification. The actual output signals *P*_bef_, *P*_aft_, *Q*_bef_, and *Q*_aft_ are calculated based on the CFD model.

The initial input pressure (i.e., *U*_F_) was set as 1.5 × 10^4^ Pa, corresponding to a 60% occlusion degree of the IVC blood flow. During the experiments, *U*_F_ increased gradually from *U*_F,0_ = 1.5 × 10^4^ Pa to *U*_F,∞_ = 3.0 × 10^4^ Pa, and remains constant afterward.

For comparison, two different types of input signals were tested in each experiment:

(i) The non-optimal input signal based on the sigmoid function (referred to as the non-optimal input);
(ii) The optimal input signals that are solved by Equation (12) with the selected parameters q, λ (referred to as the optimal input).

The different types of output signals shown in the experiment result figures include:

(i) The output signals of the CFD model when loading the non-optimal input signal (referred to as non-optimal CFD output);
(ii) The expected reference trajectory designed in Section 2.6 (referred to as reference trajectory);
(iii) The output signals of the CFD model when loading the optimal input signal (referred to as optimal CFD output).

Five experiments were conducted under different input signals, including the non-optimal input and the optimal input based on the four different selections of the parameters (q, λ), as outlined in Table 3.

**Table 3:** The parameter settings and the overshoot of *P*_aft_ in each experiment.

Experiment No.	Input Signal Type	Parameter	Figure	Overshoot/%
1	Non-optimal input	None	Figure 6 (non-optimal baseline, all columns); Figure 7	45.82
2	Optimal input	q = [0, 1, 0, 0], λ = 0	Figure 6, column 1	24.04
3	Optimal input	q = [0, 1, 0, 0], λ = 0.1	Figure 6, column 2; Figure 7	6.05
4	Optimal input	q = [1/6, 1/2, 1/6, 1/6], λ = 0	Figure 6, column 3	16.14
5	Optimal input	q = [1/6, 1/2, 1/6, 1/6], λ = 0.1	Figure 6, column 4	12.84

**Figure 7:**
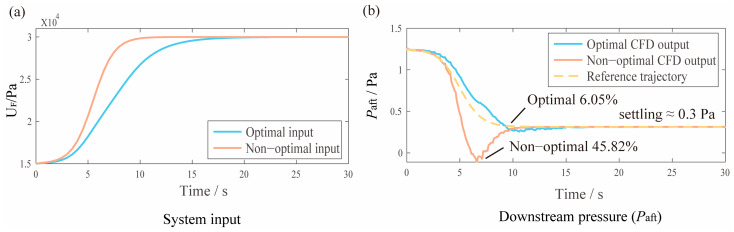
Selected optimal setting (q = [0, 1, 0, 0], λ = 0.1) versus the non-optimal baseline: (**a**) system input UF and (**b**) downstream pressure Paft. Blue, optimal; orange, non-optimal baseline; yellow dashed line in (**b**), reference trajectory. The optimal input lowers the *P*_aft_ overshoot from 45.82% to 6.05%.

In the experiments, q = [0, 1, 0, 0] means only considering the error between output signal Paft and its reference trajectory (i.e., the second subsystem) in the objective function. λ = 0 means that the penalty on the amplitude variation in the input signal *U*_F_ is not considered.

**Figure 8:**
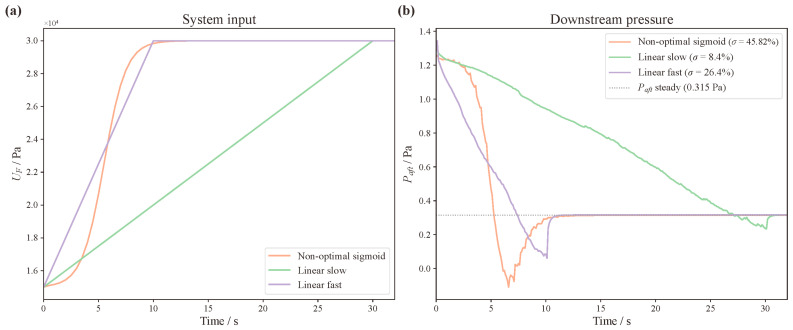
Two constant-rate linear inflation ramps versus the non-optimal sigmoid baseline: (**a**) system input *U*_F_ and (**b**) downstream pressure *P*_aft_. Profiles: non-optimal sigmoid (orange), slow linear ramp to *U*_F_ = 3.0 × 10^4^ Pa over about 30 s (sage green), and fast linear ramp to the same target over about 10 s (lavender); dotted line in (**b**), settled *P*_aft_ (about 0.315 Pa). Overshoot *σ*, defined as in Figure 7: 45.82% (sigmoid), 8.4% (slow), 26.4% (fast). Both ramps reduce the overshoot only by slowing inflation.

To benchmark the proposed model-based design against simple inflation-shaping heuristics, two additional heuristic inputs were applied to the same CFD model: a slow constant-rate linear ramp that raised *U*_F_ from 1.5 × 10^4^ to 3.0 × 10^4^ Pa over about 30 s, and a fast constant-rate linear ramp that reached the same target over about 10 s. These heuristic inputs were evaluated based on the downstream pressure overshoot and compared against both the non-optimal sigmoid baseline and the optimally designed input signal (Section 2, Figure 8).

## 3. Results

The input and output responses of the four optimal settings, each compared against the common non-optimal baseline, are organized in Figure 6 as a three-row by four-column array. Rows (a), (b), and (c) show the balloon input pressure *U*_F_, the downstream pressure *P*_aft_, and the downstream flow rate *Q*_aft_, respectively, and the four columns correspond, from left to right, to q = [0, 1, 0, 0], λ = 0; q = [0, 1, 0, 0], λ = 0.1; q = [1/6, 1/2, 1/6, 1/6], λ = 0; and q = [1/6, 1/2, 1/6, 1/6], λ = 0.1, with the selected setting boxed in the second column. The non-optimal baseline (orange) is reproduced in every column for reference. The upstream responses *P*_bef_ and *Q*_bef_, are reported across the same four settings in Figures S3 and S4. Blue and orange solid lines denote the optimal and non-optimal cases, and the yellow dashed lines in rows (b) and (c) the reference trajectories. The downstream pressure overshoot, the primary penalized objective, is shown for the recommended setting (q = [0, 1, 0, 0], λ = 0.1) in Figure 7, which presents the input pressure *U*_F_ in panel (a) and the downstream pressure *P*_aft_ in panel (b): the non-optimal sigmoid inflation produces a 45.82% overshoot in *P*_aft_, whereas the optimized input reduces it to 6.05%, a reduction of 39.77 percentage points, while preserving effective occlusion.

In addition to the output curves, the overshoots of the output signal *P*_aft_ in each experiment are listed in Table 3, which are calculated as [39]: *σ* = (*y*_m_ − *y*_∞_)/(*y*_∞_ − *y*_0_) × 100%, where *y*_m_, *y*_∞_, and *y*_0_ respectively represent the maximum value, final value, and initial value of the output signal.

To benchmark the proposed model-based input design, two simple constant-rate linear inflation ramps were compared against the non-optimal sigmoid baseline to demonstrate their limitations (Figure 8). A slow ramp reaching *U*_F_ = 3.0 × 10^4^ Pa over about 30 s reduced the downstream pressure overshoot to 8.4%, and a fast ramp reaching the same target over about 10 s reduced it to 26.4%, against 45.82% for the sigmoid baseline. Both heuristics lowered the overshoot only by slowing inflation: the slow ramp attained the smallest heuristic overshoot at roughly three times the inflation time, whereas the fast ramp, at an inflation time comparable to the optimally designed input, left a markedly larger overshoot. The optimally designed input reduced the overshoot further, to 6.05% (Table 3), while preserving a short settling time, so neither open-loop heuristic matched the model-based design on the overshoot-versus-speed trade-off.

Although direct in vivo measurements of dynamic balloon-induced infra-hepatic IVC pressure transients remain scarce, prior clinical studies of IVC balloon occlusion have documented that rapid inflation produces hemodynamic perturbations on a timescale of seconds. In a prospective cohort of 20 patients undergoing thoracic endovascular aortic repair, balloon occlusion of right atrial inflow at the suprahepatic IVC level lowered mean arterial pressure to 50 mmHg within a median of 43 s, with parameters returning to baseline within 42 s after deflation [40]. These observations also frame the pressure envelope within which a future device would operate: controlled low central venous pressure during hepatic resection is commonly targeted at 5 cmH_2_O or below (approximately 3.7 mmHg) to limit blood loss [41], whereas mean arterial pressure is generally maintained at or above 65 mmHg to avoid organ hypoperfusion [42]. The present in silico results do not operate within these clinical limits; they are stated here as the clinically meaningful thresholds that the proposed framework would be required to respect once translated. Experimental porcine work has further shown that proximal resuscitative endovascular balloon occlusion of the vena cava (REBOVC) alone causes severely decreased systemic blood pressure and cardiac output, requiring termination within 5 min when used without concomitant aortic occlusion [16]. Although these clinical and experimental observations focus on systemic hemodynamics rather than local downstream IVC pressure transients, the seconds-to-minutes timescale of the documented perturbations is consistent with the transient pressure events predicted by the present in silico framework, supporting the clinical relevance of input design that limits downstream pressure overshoot during balloon-based vascular occlusion.

## 4. Discussion

### 4.1. Effect of MPC Parameters on Hemodynamic Response

From the simulation results, we have the following observations.

(1) The advantages of using the optimal input signals: Table 3 shows that, relative to the non-optimal input, the optimal input reduces the *P*_aft_ overshoot for all settings. The largest reduction is obtained at the recommended setting q = [0, 1, 0, 0], λ = 0.1, where the overshoot falls to 6.05%. The contributions of the two weights to this reduction are examined next.
(2) The functions of the parameter λ: Comparing the first and second columns of Figure 6, where q is fixed at [0, 1, 0, 0] and only λ changes, the optimal input and the CFD outputs are smoother at λ = 0.1 than at λ = 0. The same contrast holds between the third and fourth columns, where q = [1/6, 1/2, 1/6, 1/6]. The amplitude-variation penalty λ therefore produces a smoother optimal input *U*_F_ and smoother output responses.
(3) The effect of the tracking weight q: At λ = 0 (first and third columns of Figure 6, with the upstream responses *P*_bef_ and *Q*_bef_ in the corresponding columns of Figures S3 and S4), the optimal *P*_bef_, *Q*_bef_, and *Q*_aft_ lag slightly behind their reference trajectories and the tracking of *P*_aft_ is poor. At λ = 0.1, comparing the second and fourth columns shows that *P*_aft_ for q = [0, 1, 0, 0] (second column, row (b)) is smoother and overshoots less than for q = [1/6, 1/2, 1/6, 1/6] (fourth column). Choosing q = [1/6, 1/2, 1/6, 1/6] accelerates the responses of *P*_bef_, *Q*_bef_, and *Q*_aft_ (Figures S3 and S4 and row (c) of Figure 6) relative to q = [0, 1, 0, 0], at the cost of larger overshoot and fluctuation in *P*_aft_. The weight q therefore governs a trade-off between faster *P*_bef_, *Q*_bef_, and *Q*_aft_ responses and smaller *P*_aft_ overshoot. We adopt q = [0, 1, 0, 0] and λ = 0.1 as the preferred setting.

### 4.2. Significance of the Proposed Control Framework

Based on the preceding analysis, a re-evaluation of our control framework is warranted. While it may be intuitive that decelerating the balloon inflation rate can mitigate output signal overshoot, this heuristic approach fails to determine what inflation rate is optimal. It also does not provide a quantitative method for managing the inherent trade-off between occlusion speed and pressure safety. The heuristic comparison in Section 3 (Figure 8) confirms this: two constant-rate linear inflation ramps reduce the overshoot only by slowing inflation, and neither matches the overshoot achieved by the optimally designed input at a comparable inflation time.

The optimal input generation framework proposed herein, which is based on MPC theory, offers a systematic and reproducible methodology. It generates an input trajectory that trades off downstream pressure tracking against input smoothness, with the relative weighting set by the cost-function parameters q and λ rather than by hard inequality constraints. For the selected setting q = [0, 1, 0, 0], the cost penalizes only the downstream pressure (subsystem 2), which directly addresses the clinical gap identified earlier: the absence of a quantitative basis for balancing occlusion efficacy against downstream pressure overshoot. This work establishes a methodological foundation for subsequent in vitro experiments and later clinical evaluation. Mechanical occlusion is one of several approaches to intraoperative and peripheral blood-flow regulation; non-invasive thermal methods such as far-infrared stimulation have also been explored for modulating peripheral circulation [43].

### 4.3. Clinical Implementation Considerations

A real-time implementation of the proposed input-design strategy would require real-time pressure feedback from the regions proximal and distal to the balloon. Such measurements are clinically feasible using existing catheter-based sensing technologies. Pressure-sensing guidewires and multi-lumen catheters equipped with micro-tip transducers are routinely employed in cardiovascular interventions, including coronary fractional flow reserve (FFR) assessment and pulmonary artery pressure monitoring [44,45]. Similar sensing principles can be incorporated into a balloon-occlusion catheter, allowing simultaneous acquisition of *P*_bef_ and *P*_aft_ during inflation without increasing procedural complexity. In this study, ideal pressure measurements are assumed to isolate the control-design problem; future development may also explore observer-based estimation strategies to reduce reliance on full-state sensing while maintaining safety performance.

A pragmatic translation pathway involves implementing the proposed algorithm as a hardware–software augmentation of existing FDA-cleared balloon–catheter platforms rather than developing a de novo device. Aortic balloon occlusion catheters, exemplified by the ER-REBOA Plus catheter (Prytime Medical, San Antonio, TX, USA), have received FDA 510 (k) clearance for resuscitative endovascular balloon occlusion of the aorta, illustrating an established regulatory route for compliant low-pressure occlusion balloons of comparable geometry. Endovascular balloon occlusion of the vena cava has likewise been investigated in clinical and experimental settings [13,16]. The Swan–Ganz pulmonary artery catheter family (Edwards Lifesciences, Irvine, CA, USA) provides a corresponding design precedent for multi-lumen catheters that integrate pressure transducers with a balloon-tipped distal segment. Adaptation to infra-hepatic IVC occlusion would require (i) repositioning the pressure-sensing ports so that *P*_bef_ and *P*_aft_ are sampled across the balloon, (ii) integrating an electromechanical or pneumatic actuator capable of executing the MPC-derived inflation trajectory at the millisecond timescale of the predictive horizon, and (iii) embedding the optimization solver in firmware that respects the bandwidth and latency constraints of an intraoperative system. Recent advances in fiber-optic Fabry–Perot pressure sensors and micro-electromechanical (MEMS) transducers offer compact, drift-resistant options for catheter-based deployment, and several of these sensing modalities have already been incorporated into FDA-cleared interventional devices.

Regulatory translation of the proposed framework would follow established pathways for combination devices in which a hardware platform (the catheter and actuator) is coupled with a software-based control algorithm. The control software constitutes Software as a Medical Device (SaMD) under the FDA’s Total Product Lifecycle (TPLC) framework, and IEC 62304 would classify it as the highest software safety class (Class C), since erroneous actuation could compromise hemodynamic stability. Verification and validation activities would accordingly include benchtop testing under ASME V&V 40 credibility-assessment principles [46] and the corresponding FDA risk-informed credibility framework for computational modeling in medical device submissions [47], biocompatibility evaluation per ISO 10993 [48], and risk management per ISO 14971, complementing the ex vivo and in vivo studies discussed in Section 6. The present formulation employs a fixed, non-learning optimal input signal with prespecified weighting matrices, which could, once the method is matured and validated, support 510 (k) clearance via substantial equivalence to predicate balloon-occlusion devices, supported by software documentation under 21 CFR Part 820 Quality System Regulation. Should subsequent iterations incorporate adaptive system identification or patient-specific parameter updating, the FDA’s Predetermined Change Control Plan (PCCP) guidance for AI/ML-enabled medical devices, finalized in December 2024, would provide a structured mechanism for governing post-market algorithm modifications without repeated premarket submissions. These regulatory considerations describe a route the method could follow rather than a status it has reached. The present study is an early-phase in silico investigation, and the control algorithm will require benchtop, ex vivo, and in vivo evaluation, followed by clinical testing, before any regulatory pathway becomes applicable; mapping the development effort to these milestones early is intended only to guide that later work.

## 5. Conclusions

This study presents a control-oriented computational framework for regulating downstream pressure overshoot during balloon occlusion of the infra-hepatic inferior vena cava. CFD-FSI simulations of conventional inflation revealed a 45.82% downstream pressure overshoot relative to baseline, identifying *P*_aft_ as the dominant transient that conventional fixed-rate inflation cannot suppress. A model-based optimal input design strategy was developed using reduced-order dynamic models identified from CFD-generated data. With optimal weighting parameters *q* = [0, 1, 0, 0] and λ = 0.1, the proposed optimal input design method reduced downstream pressure overshoot from 45.82% to 6.05% (a reduction of 39.77 percentage points) while preserving >90% flow occlusion at *U*_F_ = 3 × 10^4^ Pa. These results demonstrate that systematic input design can reduce downstream pressure overshoot while preserving occlusion efficacy, an outcome that conventional inflation profiles fail to achieve. The present work constitutes a first-stage in silico investigation; translation requires staged benchtop, ex vivo, and in vivo validation, together with subsequent clinical testing, as outlined in Section 6; the present results provide only the methodological basis for this staged pathway, and the regulatory considerations in Section 4.3 describe a route the method could follow once matured rather than one already established here. Future modeling work will incorporate compliant venous walls, patient-specific geometries, and physiologically realistic pulsatile boundary conditions to further enhance translational fidelity and to support eventual integration with FDA-cleared catheter platforms in liver-resection applications.

## 6. Limitations and Future Work

The present study has completed numerical verification through grid-independence analysis (GCI of 0.66%, Section 2.3.5), time-step independence (Figure S1), Reynolds-number verification confirming laminar flow at Re ≈ 254 (Section 2.3.4), and out-of-sample validation of the identified ARX models (Figure S2). Physical validation against benchtop, ex vivo, or in vivo measurements has not been performed. We assess this status under the risk-informed credibility framework of ASME V&V 40 [46], which holds that verification, validation, and uncertainty quantification activities should be commensurate with the risk of the intended use of the model, defined under the context of use (COU). Morrison et al. [49] applied this framework to a cardiovascular CFD case, showing that the required level of validation scales with whether the model is used to motivate a candidate design or to certify device performance. The COU of the present study is the methodological evaluation of a model-based optimal input signal design strategy intended to motivate a downstream-pressure-aware control signal design architecture, rather than to certify absolute in vivo pressure values. Under this COU, numerical verification combined with qualitative reproduction of the relevant hemodynamic phenomenon provides credibility commensurate with the proof-of-concept risk level; absolute-magnitude calibration is deferred to the staged validation described below.

The qualitative phenomenon central to this work, a transient downstream-pressure excursion following rapid balloon inflation, is independently documented in clinical and experimental settings. A contemporary narrative review reports that IVC balloon occlusion can produce a rapid reduction in systemic blood pressure to approximately 50 mmHg within 15 to 90 s [50]. In a live porcine model of percutaneous double-balloon IVC occlusion, Iwashita et al. recorded a 20 mmHg reduction in mean arterial pressure attributable to the occlusion [51]. Together with the clinical [40] and experimental [16] observations cited earlier, these reports independently support the reality and clinical relevance of the modeled phenomenon, which the present in silico predictions reproduce qualitatively. Absolute-magnitude calibration against physiological conditions is reserved for subsequent validation stages; the conclusion that model-based input shaping suppresses the simulated overshoot does not depend on this calibration, since the optimization operates on the response shape rather than on absolute pressure levels.

Several specific modeling assumptions warrant explicit acknowledgement. First, the IVC wall is modeled as rigid, whereas physiological venous tissue exhibits compliance; the simulated overshoot therefore represents an upper bound, and the ability of the control signal to suppress it under this worst-case scenario translates into a protective safety margin. Second, the inlet-outlet boundary conditions adopt a steady inflow representative of an averaged intraoperative state and do not resolve respiratory- or cardiac-induced pulsatility. Third, the simulations were conducted at a low-pressure operating point selected for numerical stability and methodological demonstration; the reported overshoot behavior is specific to this operating point and to the present grid resolution, and confirmation at physiological scale is left to subsequent stages. Fourth, the third-order Ogden hyperelastic parameters were obtained from the ANSYS Mechanical material library rather than from device-specific mechanical testing, introducing a known uncertainty in the absolute force-displacement relationship at the balloon wall. Fifth, physical validation against benchtop measurements, ex vivo porcine preparations, or in vivo recordings has not been performed; the credibility of the model for its intended use rests on the verification activities, on the qualitative agreement with the independent hemodynamic evidence summarized above, and on the credibility framework outlined in the first paragraph of this section.

This staged validation strategy aligns with the risk-informed credibility framework introduced above, progressively closing the credibility gap as the COU is extended toward in vivo decision-relevant use. The first stage uses silicone vascular phantoms of the infra-hepatic IVC under controlled pressure-flow boundary conditions to deliver a first quantitative comparison between simulated and measured *P*_aft_ under controlled reference conditions, supporting solver validation and parameter sensitivity analysis. The second stage uses ex vivo fresh porcine IVC preparations to quantify how venous-wall compliance and viscoelasticity modulate the downstream-pressure overshoot relative to the rigid-wall upper bound of the present study, and enables acute repeatable testing of the proposed optimal input without confounding from systemic hemodynamics or anesthesia. The third stage involves in vivo validation in porcine hepatectomy models under fully physiological conditions; outcome measures will include peak *P*_aft_ excursion, time-to-target flow occlusion, hemodynamic stability metrics (mean arterial pressure, central venous pressure, arrhythmia incidence), and post-procedural hepatic (ALT, AST, bilirubin) and renal (creatinine) function markers. Each stage contributes credibility evidence at a level of physical realism matched to a successively higher-risk COU, and together they would support the regulatory pathway outlined in Section 4.3.

Beyond experimental validation, several modeling extensions warrant future work. Alternative reduced-order FSI formulations, such as lumped-parameter or differential-algebraic representations of the balloon–vessel interaction, may accelerate the control algorithm development cycle. Patient-specific vascular geometries derived from clinical imaging, together with image-based boundary conditions, would enhance physiological fidelity and enable individualized input signal optimization. Incorporation of compliant vessel walls and pulsatile inflow into the FSI model would quantify the overshoot attenuation contributed by venous compliance and allow direct comparison against the rigid-wall upper bound presented here. The present optimal input signal design method penalizes downstream-pressure overshoot through a weighted cost function and imposes no hard inequality constraints; once clinically meaningful pressure thresholds are defined through the validation stages above, the same formulation can be extended to a constrained, optimization-based MPC, supporting a transition from the proof-of-concept role of the present study to deployable control software intended for translational and ultimately certified use.

## Figures and Tables

**Table 1:** Mesh independence verification: downstream pressure *P*_aft_ at full balloon expansion and grid convergence index (GCI) at three mesh densities.

Mesh	Number of Elements	*P*_aft_ (Pa)	GCI (%)
Coarse	1.16 × 10^5^	0.494	N/A
Medium	3.34 × 10^5^	0.412	4.73
Fine	1.03 × 10^6^	0.399	0.66

Note: Since GCI evaluates the discretization uncertainty of a given mesh relative to a coarser one, N/A denotes not applicable for the coarse mesh as it serves as the baseline.

**Table 2:** Parameter identification results, training-set NRMSE, and out-of-sample validation NRMSE and R^2^ of the control models.

Subsystem	Output Variable	*θ*_*i*,1_	*θ*_*i*,2_	*θ*_*i*,3_	*θ*_*i*,4_	Training NRMSE	Validation NRMSE	Validation R^2^
1	*P*_bef_	−1.6713	0.7859	1.6353	−1.0591	0.0405	0.1326	0.9824
2	*P*_aft_	−1.6723	0.8565	−0.3144	0.2559	0.1164	0.2381	0.9433
3	*Q*_bef_	−1.7409	0.8163	−0.0024	0.0016	0.0363	0.1224	0.9850
4	*Q*_aft_	−1.6968	0.7767	−0.0028	0.0021	0.0374	0.1264	0.9840

## Data Availability

The data and code supporting this study have been deposited at Zenodo (https://doi.org/10.5281/zenodo.20204214) and will be made publicly accessible upon acceptance of the manuscript. Complete ANSYS Workbench project files are available from the corresponding author upon reasonable request.

## Supplementary Materials

The following supporting information can be downloaded at: https://www.mdpi.com/article/10.3390/bioengineering13070749/s1. Figure S1: Time-step independence of the peak transient downstream pressure. (A) Transient *P*_aft_ during inflation at solver time steps of 0.02, 0.01, and 0.005 s; the three traces are nearly indistinguishable. (B) Zoom on the inflation-induced trough; the peak *P*_aft_ is −6.424, −6.443, and −6.459 Pa at 0.02, 0.01, and 0.005 s, a maximum spread below 0.9%, confirming that the 0.01 s production step resolves the peak. Figure S2: Out-of-sample validation of the identified ARX models. A sinusoidal input distinct from the identification M-sequence was applied to the same coupled FSI CFD model, and the one-step-ahead ARX prediction (red dotted) is compared with the CFD response (black solid) for (a) upstream pressure *P*_bef_, (b) downstream pressure *P*_aft_, (c) upstream mass flow *Q*_bef_, and (d) downstream mass flow *Q*_aft_. The one-step-ahead R^2^ exceeds 0.94 for all four subsystems (Table 2), with *P*_aft_ the most demanding. Figure S3: Upstream pressure P_bef_ under the four optimal input settings. From left to right, the four panels correspond to q = [0, 1, 0, 0], λ = 0; q = [0, 1, 0, 0], λ = 0.1; q = [1/6, 1/2, 1/6, 1/6], λ = 0; and q = [1/6, 1/2, 1/6, 1/6], λ = 0.1, in the same order as the columns of Figure 6, and the second panel (q = [0, 1, 0, 0], λ = 0.1) is the selected optimal setting. In each panel, the blue and orange solid lines denote the CFD upstream pressure under the optimal and non-optimal inputs, respectively, and the yellow dashed line denotes the reference trajectory. *P*_bef_ is not penalized in the cost function and is reported here to complement the downstream responses shown in Figure 6. Figure S4: Upstream flow rate *Q*_bef_ under the four optimal input settings. The panel order and weight settings match Figure 6 and Figure S3: from left to right, q = [0, 1, 0, 0], λ = 0; q = [0, 1, 0, 0], λ = 0.1; q = [1/6, 1/2, 1/6, 1/6], λ = 0; and q = [1/6, 1/2, 1/6, 1/6], λ = 0.1, with the second panel (q = [0, 1, 0, 0], λ = 0.1) the selected optimal setting. In each panel, the blue and orange solid lines denote the CFD upstream flow rate under the optimal and non-optimal inputs, respectively, and the yellow dashed line denotes the reference trajectory. Like *P*_bef_, *Q*_bef_ is not penalized in the cost function and is provided here to complement the downstream responses in Figure 6.
