# Supplementary material for: Modeling and Optimal Input Design for Infra-Hepatic Blood Flow Regulation Systems
Source: Bioengineering (Basel). 2026 Jun 26;13(7):749. doi: 10.3390/bioengineering13070749 (PMC13403439; doi:10.3390/bioengineering13070749)
Supplement: Supplementary file 1 [file bioengineering-13-00749-s001.zip › bioengineering-4347601-supplementary.pdf]

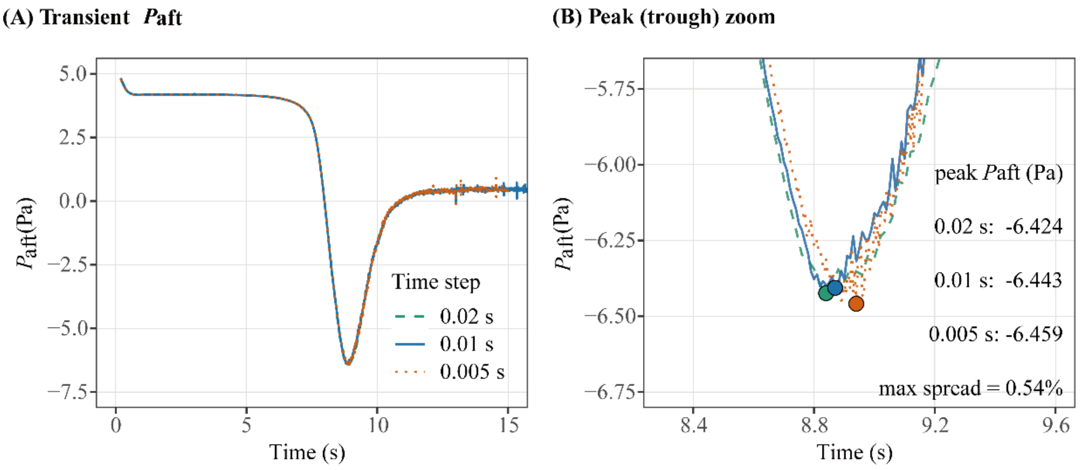

Figure S1. Time-step independence of the peak transient downstream pressure. (A) Transient *P*aft during inflation at solver time steps of 0.02, 0.01, and 0.005 s; the three traces are nearly indistinguishable. (B) Zoom on the inflation-induced trough; the peak *P*aft is −6.424, −6.443, and −6.459 Pa at 0.02, 0.01, and 0.005 s, a maximum spread below 0.9%, confirming that the 0.01 s production step resolves the peak.

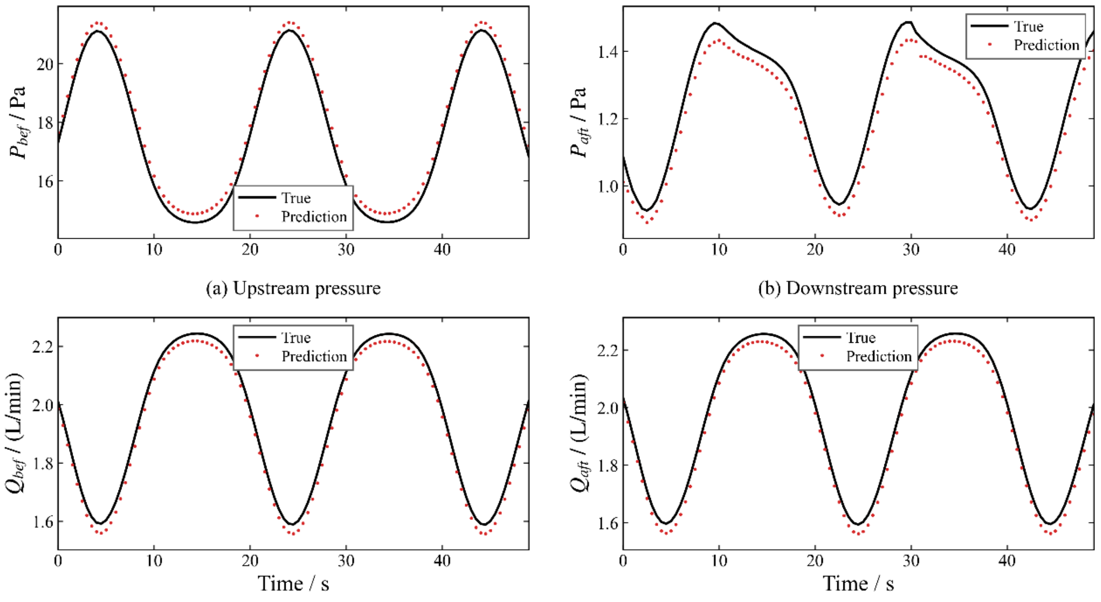

(a) Upstream pressure (b) Downstream pressure

(c) Upstream mass flow (d) Downstream mass flow

Figure S2. Out-of-sample validation of the identified ARX models. A sinusoidal input distinct from the identification M-sequence was applied to the same coupled FSI CFD model, and the one-step-ahead ARX prediction (red dotted) is compared with the CFD response (black solid) for (a) upstream pressure P_{bef}, (b) downstream pressure P_{aft}, (c) upstream mass flow Q_{bef}, and (d) downstream mass flow Q_{aft}. The one-step-ahead R^2 exceeds 0.94 for all four subsystems (Table 2), with P_{aft} the most demanding.

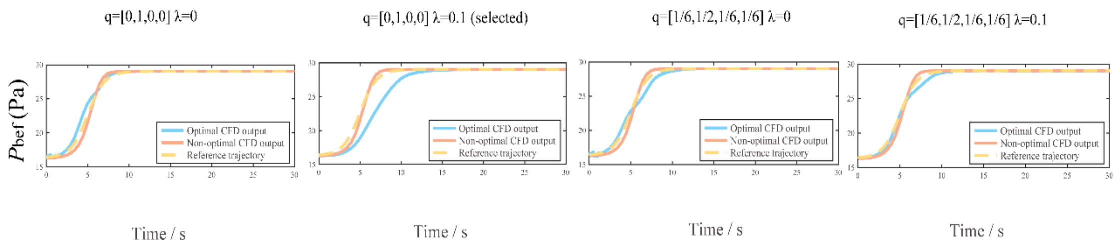

Figure S3. Upstream pressure Pbef under the four optimal input settings. From left to right, the four panels correspond to q = [0,1,0,0], λ = 0; q = [0,1,0,0], λ = 0.1; q = [1/6,1/2,1/6,1/6], λ = 0; and q = [1/6,1/2,1/6,1/6], λ = 0.1, in the same order as the columns of Figure 6, and the second panel (q = [0,1,0,0], λ = 0.1) is the selected optimal setting. In each panel, the blue and orange solid lines denote the CFD upstream pressure under the optimal and non-optimal inputs, respectively, and the yellow dashed line denotes the reference trajectory. Pbef is not penalized in the cost function and is reported here to complement the downstream responses shown in Figure 6.

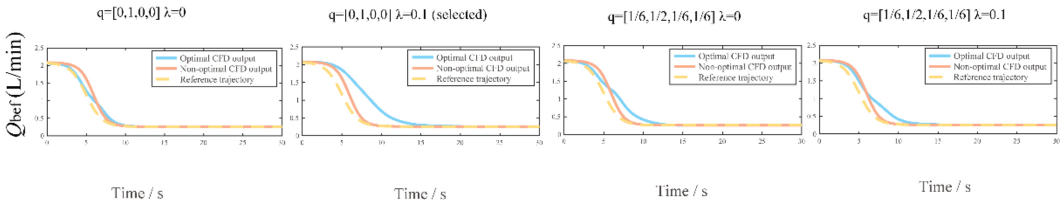

Figure S4. Upstream flow rate Qbef under the four optimal input settings. The panel order and weight settings match Figure 6 and Figure S3: from left to right, q = [0,1,0,0], λ = 0; q = [0,1,0,0], λ = 0.1; q = [1/6,1/2,1/6,1/6], λ = 0; and q = [1/6,1/2,1/6,1/6], λ = 0.1, with the second panel (q = [0,1,0,0], λ = 0.1) the selected optimal setting. In each panel, the blue and orange solid lines denote the CFD upstream flow rate under the optimal and non-optimal inputs, respectively, and the yellow dashed line denotes the reference trajectory. Like Pbef, Qbef is not penalized in the cost function and is provided here to complement the downstream responses in Figure 6.
